# Infections, Animal-Source Foods, and Micronutrient Status as Correlates of Serum IGF-1 in Children with Stunting: A Cross-sectional Study in Uganda

**DOI:** 10.1016/j.tjnut.2025.10.017

**Published:** 2025-10-13

**Authors:** Emma Madigan, Nynne Emilie Nielsen, Joseph Mbabazi, Rolland Mutumba, Christian Ritz, Suzanne Filteau, André Briend, Kim F Michaelsen, Christian Mølgaard, Ezekiel Mupere, Benedikte Grenov, Henrik Friis

**Affiliations:** 1 Department of Nutrition, Exercise and Sports, University of Copenhagen, Copenhagen, Denmark; 2 Department of Paediatrics and Child Health, School of Medicine, College of Health Sciences, Makerere University, Kampala, Uganda; 3 The National Institute of Public Health, University of Southern Denmark, Copenhagen, Denmark; 4 Department of Population Health, London School of Hygiene and Tropical Medicine, London, United Kingdom; 5 Tampere Center for Child, Adolescent and Maternal Health Research, Faculty of Medicine and Health Technology, Tampere University and Tampere University Hospital, Tampere, Finland

**Keywords:** children, stunting, IGF-1, animal-source foods, milk, vitamin A, Uganda

## Abstract

**Background:**

Serum insulin-like growth factor-1 (serum IGF-1) is important for growth in childhood. Inflammation downregulates serum IGF-1, but the roles of intake of animal-source foods and micronutrient status are not well known.

**Objectives:**

We assessed the associations of infections, intake of animal-source foods, iron, B12, folate, and vitamin A status with serum IGF-1 among stunted children.

**Methods:**

We conducted a cross-sectional study, using data from a nutrition trial among 12–59-mo-old stunted Ugandan children. Data on sociodemography, anthropometry, breastfeeding, dietary intake, and morbidity were collected. Serum IGF-1 and markers of micronutrient status and inflammation were determined. A rapid malaria test was done. Fat and fat-free mass were measured using bioimpedance. Tobit regression was used to assess correlates of serum IGF-1.

**Results:**

Of 750 children, 45.1% (*n =* 338) were girls and 29.6% (*n =* 222) were <2 y. Serum IGF-1 was available on 98.7% (*n =* 740). Median (interquartile range) serum IGF-1 was 37.4 (24.2, 53.3) μg/L, 11.8 [95% confidence interval (CI): 8.8, 14.9] μg/L lower in males, and 0.6 (95% CI: 0.5, 0.6) μg/L higher per month higher age. Inflammation markers were strong negative correlates of serum IGF-1. Positive malaria test was associated with lower serum IGF-1 (−4.7 μg/L, 95% CI: −7.8, −1.6), but not after inflammation adjustment (1.3 μg/L, 95% CI: −1.9, 4.6). Serum IGF-1 was associated with height-for-age *z* and fat-free mass. Serum retinol-binding protein <0.70 and 0.70–1.05, compared with >1.05 μmol/L was associated with 14.3 (95% CI: 9.6, 19.1) and 6.1 (95% CI: 1.6, 10.6) μg/L lower serum IGF-1 after adjustments for age, sex, and inflammation. Markers of other micronutrients were not. Intake of milk, but not meat or eggs, was associated with 3.9 (95% CI: 0.7, 7.1) μg/L higher serum IGF-1.

**Conclusions:**

Milk intake and vitamin A status were positively associated with serum IGF-1. In contrast to milk, vitamin A has not consistently been associated with growth. This requires further investigation.

## Introduction

Stunting is widespread in low-income settings and is associated with infectious disease morbidity and mortality and impaired cognitive development in childhood [1]. Stunting is associated with lower fat-free mass (FFM) [2] and possibly a higher risk of cardiometabolic diseases in later life [3].

The main determinants of stunting are low-quality diets and infections [1]. The typical diet in low-income settings in Africa is based on maize or other starch-rich staple foods, with little or no animal-source foods [4,5]. Such a diet is low in B12, preformed vitamin A, and heme-iron, only found in animal-source foods. It also has a high content of phytates, which impairs the bioavailability of nonheme iron, and growth nutrients like zinc [4] and amino acids [6]. A high burden of infectious diseases may further compromise nutritional status [7]. Diarrhea leads directly to loss of nutrients, whereas generalized infections precipitate an acute-phase response, which reduces the intake and absorption of nutrients, and increases utilization and excretion [7].

The effects of nutritional deficiencies and inflammation on childhood growth are partly mediated via the growth hormone (GH)/insulin-like growth factor-1 (IGF-1) axis [8]. GH is produced in the anterior pituitary gland, and IGF-1 is primarily produced in the liver [9]. IGF-1 is also produced in muscle and bones, where it acts locally through paracrine and autocrine signaling [8]. IGF-1 is important for the epiphyseal growth plates, where it promotes chondrocyte differentiation, proliferation, and ossification [9], and hence is important for bone and linear growth.

Inflammation is known to downregulate serum IGF-1 [10]. Breastfeeding was associated with higher serum IGF-1 among children in Burkina Faso, most likely because the alternative contained little animal-source foods [11]. Breastfeeding was associated with lower serum IGF-1 in high-income countries, where nonbreastfed children receive formula with high protein levels [12].

Intake of animal-source foods, especially milk, has been associated with higher serum IGF-1 in children [13]. Intervention trials among small children have demonstrated that zinc supplementation increases serum IGF-1 [14], and experimental zinc deficiency was shown to reduce serum IGF-1 in rats compared with pair-fed controls [15]. Deficiencies of iron, vitamin A, and B12 are widespread in low-income settings, not least among children with stunting [16,17], but are not considered to be growth nutrients [18]. However, vitamin A has been shown to be involved in GH gene expression [19], and B12 and folate are essential to DNA synthesis [20].

We have recently shown that children on a stunting trajectory gain fat at the expense of FFM [21], and that a large-quantity lipid-based nutrient supplement with a micronutrient premix increases serum IGF-1 and results in catch-up of linear growth accompanied by FFM accretion [21]. Milk protein had no additional effect on serum IGF-1 compared with high-quality soy protein. Using baseline data from the same trial, we aimed to assess the role of infections, intake of animal-source foods as well as iron, B12, folate, and vitamin A status as correlates of serum IGF-1 among children with stunting.

## Methods

### Study design, area, and population

This cross-sectional study used baseline data from the MAGNUS trial (Milk Affecting Growth, Cognition, and the Gut in Child Stunting), a randomized, community-based nutrition trial among children with stunting (ISRCTN13093195). The trial was carried out in the Jinja District, Eastern Uganda, where the prevalence of stunting was 29%, corresponding to the national average. The MAGNUS study was implemented in 2 community health centers, and the enrolled children were recruited from surrounding rural and urban communities [21,22].

Children aged 12–59 mo with length/height-for-age *z*-score (HAZ) <-2 whose caregiver had provided written informed consent were eligible. Children with severe acute malnutrition, that is weight-for-length/height *z*-score <-3, mid-upper arm circumference (MUAC) <11.5 cm, or bipedal pitting edema, were excluded and referred to a local hospital. Furthermore, children with disabilities impeding the ability to eat or length/height measurements, medical complications requiring hospitalization, allergy to milk or peanuts, or participation in another study were excluded. Families with plans to relocate from the study area could not be recruited, and only 1 child per household could participate.

### Data collection

At baseline, information on participant age and birth weight was gathered from the caregiver and birth certificates. The caretaker was interviewed and administered questionnaires containing information about sociodemographics, current breastfeeding, and intake of foods from different food groups within the last 24 h, as well as morbidity within the last 2 wk. The children underwent a clinical examination, anthropometric measurements, body composition assessment, and blood sampling.

### Anthropometry and body composition

Length/height was measured on children below/above 24 mo using a wooden ShorrBoard (Weigh and Measure). MUAC was measured to the nearest 1 mm on the left arm between the acromion and olecranon using a standard nonelastic MUAC tape. Weight was measured on a weekly calibrated digital scale (SECA 874) and recorded to the nearest 100 g. All anthropometric measurements were done in triplicate, and the median was used. Anthropometric indices were calculated using the STATA module: zscore06. Stunting was defined as a HAZ <-2, and severe stunting as a HAZ <-3. Fat mass (FM) and FFM were assessed by bioimpedance using Bodystat 500 instrumentation at a current of 50 kHz (Bodystat Ltd.). The children were placed on their backs with limbs spread apart, without a wet or soiled diaper, and wearing loose-fitting clothes. The measurements were made after 10 min of rest and were repeated 3 times in case of poor measurements, and with a minimum of 2 repetitions. The raw impedance data were collected to estimate total body water. An age-, stunting-, and population-specific equation was used to calculate FFM [23]. FM was calculated as total weight minus FFM, and corresponding height-corrected indices, fat-free mass index (FFMI) and fat mass index (FMI), were derived by dividing FFM and FM by length/height in meters squared.

### Blood sampling and biochemistry

Approximately 6.0 mL of blood was collected from the cubital vein and divided into plain serum tubes, lithium heparin tubes, and EDTA tubes. Samples were transported to a local laboratory, centrifuged at 2300 ×*g* (3500 rounds per minute) for 10 min, and frozen at −20° C. Weekly, samples were further transported to a biorepository (Integrated Biorepository of H3Africa Uganda, Makerere University) and stored at −80°C until shipment on dry ice to Europe for further analysis.

Children were tested for malaria using a malaria rapid diagnostics test (SD BIOLINE MALARIA AG PF) in the local laboratory, with blood from the EDTA tubes.

Serum IGF-1 was determined at the University of Copenhagen on an Immulite 2000 Analyzer (Siemens Healthcare, GmbH) with intra- and interassay coefficient of variation of 1.9%–4.2% and 4.2%–7.2%, respectively, and a limit of detection of 10.6 μg/L. Serum concentrations of C-reactive protein (serum CRP), α_1_-acid glycoprotein (serum AGP), ferritin (serum Fe), soluble transferrin receptor (serum sTfR), and retinol-binding protein (serum RBP) were determined using combined sandwich ELISA in the VitMin Lab [24]. The inter- and intraassay coefficients of these markers were 5%–14%. Serum CRP was used as a fast-reacting and serum AGP as a slow-reacting marker of inflammation, with the cut-offs of 2 and 10 mg/L, and 0.8 and 1.2 g/L, respectively. Serum Fe <12 μg/L defined depleted iron stores, and serum sTfR >8.3 mg/L defined tissue iron deficiency. Serum RBP <0.70 μmol/L defined vitamin A deficiency, and serum RBP 0.70–1.05 μmol/L defined marginal vitamin A status.

Plasma concentrations of cobalamin (plasma Cob) and folate (plasma Fol) were determined with an Advia Centaur CP immunoassay System (Siemens), and plasma methylmalonic acid (plasma MMA) was measured using liquid chromatography-tandem mass spectrometry on the AB SCIEX Triple Quad 550 system at Aarhus University Hospital, Denmark. Total imprecisions were 7.5% and 11.8%. Plasma Cob <148 pmol/L or plasma MMA >0.75 μmol/L was used to define low vitamin B12 status, and plasma Cob between 148 and 221 pmol/L to define marginal B12 status, whereas low folate status was defined by plasma Fol <14 nmol/L.

### Statistical analysis

Data were double-entered using Epidata 3.1 (Epidata Association), and statistical analyses performed in STATA version 14.2 (Stata). Background characteristics were presented as mean ±SD for continuous variables, and % (*n*) for categorical variables. The distribution of serum IGF-1 was assessed based on QQ-plots. Tobit regression analysis was used to assess potential correlates of serum IGF-1. Tobit regression is used when the dependent variable is censored. In this case, serum IGF-1 had a range within which measurements could be obtained, whereas other measurements were below the limit of detection. Tobit regression combines features of both linear regression and logistic regression, allowing for the estimation of slope coefficients although considering the censored nature of the data. The associations with serum IGF-1 were reported as regression coefficients (differences or slopes) with 95% confidence intervals (CIs), unadjusted, adjusted for age and sex, and adjusted for age, sex, and inflammation. In addition, mutual adjustments for FM and FFM, as well as FMI and FFMI, were carried out. Model checking was based on residual and normal probability plots. *P* values <0.05 were considered significant. To graphically describe trends in serum IGF-1 by age for boys and girls separately, best-fitting fractional polynomials were shown with corresponding 95% CIs. The best fits were based on the sum of 2 power terms where, for each term, the optimal exponent among −2, −1, −0.5, 0, 0.5, 1, 2, and 3 was chosen.

### Ethics

The MAGNUS study was conducted according to the Declaration of Helsinki's general regulations and ethical principles. Furthermore, the Makerere University School of Medicine Research Committee (#REC REF 2019-013), the Ugandan National Council of Science and Technology (SS 49270) approved the study, and the Danish National Committee of Biomedical Research Ethics (1906848) gave consultative approval. Verbal and written consent for trial participation was given by an informed caregiver of the participant in either Lusoga, Luganda, or English. All caregivers gave consent before inclusion. Caregivers were informed that their children were not growing well, and nutritional counseling was provided to all. Furthermore, children found ill were referred to the site health center for treatment if necessary.

## Results

Of 750 children enrolled, 45.1% (*n =* 338) were girls (Table 1) and the mean ±SD age was 30.1 ±11.7 mo. Overall, 13% (*n =* 95) were currently breastfed: 39% (*n =* 86) of 222 children <2 y, and 2% (*n =* 9) of 524 children >2 y. The malaria test was positive in 40% (*n =* 292) (Table 1), and the prevalence of fever, diarrhea, and cough in the last 2 wk was 58% (*n =* 436), 27% (*n =* 206), and 50% (*n =* 375).

**TABLE 1:** Background characteristics of 750 children aged 12–59 mo with stunting^1^.

	Boys (*n* = 412)	Girls (*n* = 338)
Age (mo)	30.8 ±11.2	33.5 ±12.2
0–23	32% (133)	26% (89)
24–35	35% (145)	34% (114)
36–59	33% (134)	40% (135)
Mid-upper arm circumference (cm)	14.5 ±1.2	14.4 ±1.1
<12.5	6.6% (27)	3.9% (13)
Length/height-for-age (*z*)	−3.1 ±0.8	−2.9 ±0.7
Weight-for-length/height (*z*)	−0.4 ±1.0	−0.3 ±0.9
Breastfed now	14% (59)	11% (36)
Malaria positive 2	42% (171)	36% (121)
Maternal age (y)		
<20	18% (70)	12% (37)
20–30	62% (238)	68% (210)
>30	20% (75)	20% (62)
Maternal education		
No	50% (103)	45% (145)
Primary	36% (141)	38% (123)
Secondary or more	14% (56)	17% (55)
Rural residence	47% (195)	41% (140)
Own livestock	49% (201)	53% (178)
Serum C-reactive protein (mg/L)		
<2	51% (207)	57% (189)
2–10	26% (106)	23% (76)
>10	23% (94)	20% (69)
Serum α_1_-acid glycoprotein (g/L)		
<0.8	18% (72)	20% (67)
0.8–1.2	29% (118)	34% (114)
>1.2	53% (217)	46% (153)

1 Data shown are mean ±SD or % (*n*). *n* may not sum up to 750 due to missing data. Only age and height-for-age z were different between sexes, *P* < 0.05.

2 Rapid diagnostic antigen test.

**FIGURE 1:**
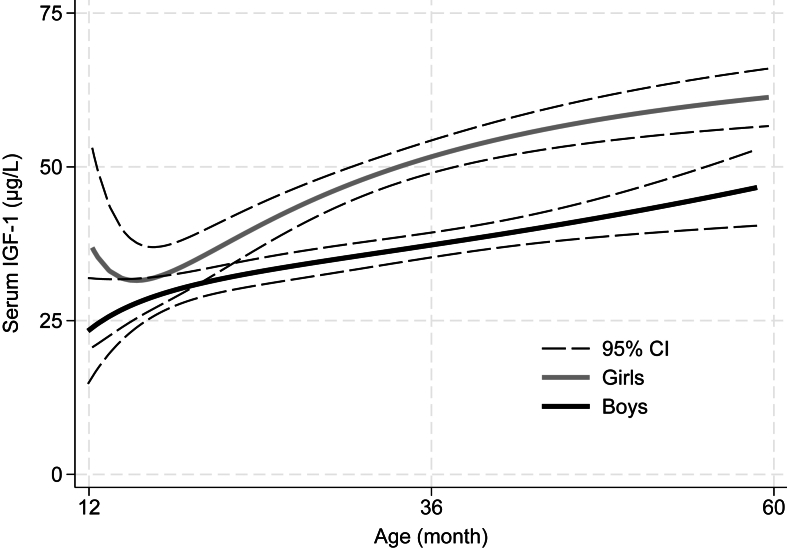
Serum insulin-like growth factor-1 (IGF-1) by age and sex among 740 children aged 12–59 mo with stunting. Data are presented as fractional polynomials with 95% confidence intervals (CIs).

Data on serum IGF-1 were available for 98.7% (*n =* 740) of the 750 children. Of these, 7.6% (*n =* 56) had values below the limit of detection (10.6 μg/L). Median (IQR) serum IGF-1 was 37.4 (24.2; 53.3) μg/L. As seen in Figure 1, after an early nadir in serum IGF-1 among girls, the increase with age was almost linear, with similar slopes for both sexes. Boys had 11.8 (95% CI: 8.8, 14.2) μg/L lower serum IGF-1 compared with females, after adjustment for age, and serum IGF-1 increased by 0.6 μg/L (95% CI: 0.5, 0.7) per month higher age, after adjustment for sex.

Although all children were stunted, severe stunting was associated with 7.0 (95% CI: 4.0, 10.0) μg/L lower serum IGF-1 after age and sex adjustments, and further adjustment for inflammation reduced the difference (−5.1, 95% CI: −8.0, −2.1) (Table 2). A positive malaria test was also associated with lower serum IGF-1 (−4.7, 95% CI: −7.8, −1.6), but the association disappeared with adjustment for inflammation (1.1, 95% CI: −2.1, 4.4). Diarrhea during the last 2 wk and on the day of examination was associated with 4.4 (95% CI: 0.7, 8.0) and 9.6 (95% CI: 2.6, 16.5) μg/L lower serum IGF-1, respectively. The association between current diarrhea and serum IGF-1 was maintained after adjustment for inflammation. Reported fever on the day of examination, but not in the previous 2 wk, was also associated with lower serum IGF-1, but was explained by inflammation. Cough was not associated with serum IGF-1. Elevated serum CRP and serum AGP were associated with lower serum IGF-1. Half of the children had serum AGP >1.2 mg/L, which was associated with 17.4 (95% CI: 13.5, 21.3) μg/L lower serum IGF-1.

**TABLE 2:** Anthropometry and infections as correlates of serum insulin-like growth factor-1 (μg/L) among 740 children aged 12–59 mo with stunting^1^.

	Age and sex adjusted^2^			Age, sex, and inflammation adjusted^3^		
	B	95% CI	*P*	B	95% CI	*P*
Height-for-age (*z*)						
≥−3 to <−2 (*n =* 425)	—	—	—	—	—	—
<−3 (*n =* 315)	−7.0	(−10.0, −4.0)	<0.001	−5.1	(−8.0, −2.1)	0.001
Weight-for-height (*z*)						
>−2 (*n =* 700)	—	—	—	—	—	—
≤−2 (*n =* 39)	−5.1	(−11.9, 1.7)	0.14	−5.3	(−11.8, 1.2)	0.11
Mid-upper arm circumference (cm)						
>12.5 (*n =* 700)	—	—	—	—	—	—
<12.5 (*n =* 40)	−7.1	(−14.0, −0.2)	0.04	−5.8	(−12.3, 0.7)	0.08
Malaria^1^						
No (*n =* 439)	—	—	—	—	—	—
Yes (*n =* 291)	−4.7	(−7.8, −1.6)	0.003	1.1	(−2.1, 4.4)	0.50
Fever <14 d						
No (*n =* 310)	—	—	—	—	—	—
Yes, not today (*n =* 359)	−1.9	(−5.1, 1.2)	0.23	1.1	(−2.0, 4.2)	0.47
Today (*n =* 71)	−9.6	(−14.9, −4.2)	0.001	−3.6	(−9.1, 1.9)	0.20
Diarrhea <14 d						
No (*n =* 539)	—	—	—	—	—	—
Yes, not today (*n =* 163)	−4.4	(−8.0, −0.7)	0.02	−2.4	(−5.9, 1.1)	0.18
Today (*n =* 38)	−9.6	(−16.5, −2.6)	0.007	−7.9	(−14.7, −1.2)	0.02
Cough <14 d						
No (*n =* 371)	—	—	—	—	—	—
Yes, not today (*n =* 105)	1.2	(−3.3, 5.7)	0.60	2.8	(−1.5, 7.1)	0.20
Today (*n =* 264)	−3.0	(−6.2, 0.3)	0.08	−0.2	(−3.4, 3.0)	0.92
Serum C-reactive protein (mg/L)						
<2 (*n =* 392)	—	—	—	—	—	—
2–10 (*n =* 181)	−5.2	(−9.9, −0.5)	0.03	−0.1	(−4.8, 4.7)	0.98
>10 (*n =* 161)	−10.0	(−13.7, −6.4)	<0.001	−3.0	(−7.0, 1.0)	0.15
Serum α_1_-acid glycoprotein (g/L)						
<0.8 (*n =* 136)	—	—	—	—	—	—
0.8–1.2 (*n =* 232)	−8.0	(−12.2, −3.8)	<0.001	−7.8	(−12.0, −3.6)	<0.001
>1.2 (*n =* 370)	−17.4	(−21.3, −13.5)	<0.001	−16.2	(−20.6, −11.9)	<0.001

1 Data shown are the coefficient B with 95% confidence interval (CI) and *P* value based on Tobit regression. *n* may not sum up to 740 due to missing data.

2 Adjusted for age (continuous) and sex.

3 Adjusted for age (continuous), sex, and elevated serum C-reactive protein (CRP) and α_1_-acid glycoprotein (AGP), categorized as shown. For serum CRP and AGP, the *P* for linear trend were 0.031 and <0.001.

Both FM and FFM were associated with serum IGF-1 after adjustment for age and sex, as well as inflammation (Table 3). With FM and FFM included in the same model, both were positively associated with serum IGF-1. As can be seen from the nonoverlapping CIs, FFM was a stronger correlate of serum IGF-1 (9.8 μg/L, 95% CI: 6.9, 12.8 compared with 2.2 μg/L, 95% CI: 0.4, 4.0). The same analyses were done with FM and FFM indexed for height. Both remained associated, but the difference in magnitude of association disappeared (3.7 μg/L, 95% CI: 1.1, 6.4 compared with 2.7 μg/L, 95% CI: 1.5, 4.0).

**TABLE 3:** Body composition as correlates of serum insulin-like growth factor-1 (μg/L) among 740 children aged 12–59 mo with stunting^1^.

	Age and sex adjusted^2^			Age, sex, and inflammation adjusted^3^			Age, sex, inflammation, and mutually adjusted^4^		
	*B*	(95% CI)	*P*	*B*	(95% CI)	*P*	*B*	(95% CI)	*P*
Fat mass (kg)	3.8	(2.0, 5.7)	<0.001	4.5	(2.7, 6.2)	<0.001	2.2	(0.4, 4.0)	0.02
Fat-free mass (kg)	13.6	(10.9, 16.2)	<0.001	11.1	(8.4, 13.8)	<0.001	9.8	(6.9, 12.8)	<0.001
Fat mass index (kg/m^2^)	2.3	(0.9, 3.6)	<0.001	2.9	(1.6, 4.1)	<0.001	2.7	(1.5, 4.0)	<0.001
Fat-free mass index (kg/m^2^)	5.5	(2.8, 8.2)	<0.001	4.2	(1.5, 6.8)	0.002	3.7	(1.1, 6.4)	0.005

1 Body composition was measured using bioimpedance analysis. Data shown are coefficient B with 95% confidence interval (CI) and *P* value based on Tobit regression.

2 Adjusted for age (continuous) and sex.

3 Adjusted for age (continuous), sex, and elevated serum C-reactive protein and α_1_-acid glycoprotein (categorical).

4 In addition to adjustment for age, sex, and inflammation, the model with fat mass is adjusted for fat-free mass and vice versa.

Breastfeeding was not associated with serum IGF-1 after adjustments (Table 4). Approximately one-third of the children (30.3%, *n =* 223) had milk or milk products within the last 24 h, of which 97.3% (*n =* 217) had whole cow milk, and the remainder other milk products. Intake of milk was associated with 3.9 (95% CI: 0.7, 7.1) μg/L higher serum IGF-1, after adjustment for age, sex, and inflammation (Table 4). There was no difference in HAZ among those who had consumed milk compared with those who had not. Neither intake of meat nor eggs was associated with serum IGF-1. In a model with all of milk, meat, and egg intake, as well as age, sex, and inflammation, milk (3.9, 95% CI: 0.2, 7.1) remained associated with serum IGF-1, but meat (0.5 μg/L, 95% CI: −2.4, 3.4) and egg (−0.1 μg/L, 95% CI: −5.4, 5.2) intake were not.

**TABLE 4:** Breastfeeding and intake from animal-source foods within *the* previous 24 h as correlates of serum insulin-like growth factor-1 (μg/L) among 740 children aged 12–59 mo with stunting^1^.

	Age and sex adjusted^2^			Age, sex, and inflammation adjusted^3^		
	B	(95% CI)	*P*	B	(95% CI)	*P*
Breastfeeding						
No (*n =* 643)	—	—	—	—	—	—
Yes (*n =* 93)	0.4	(−4.7, 5.6)	0.88	−1.6	(−6.6, 3.4)	0.53
Animal-source food <24 h						
Milk						
No (*n =* 517)	—	—		—	—	
Yes (*n =* 223)	4.7	(1.4, 8.0)	0.005	3.9	(0.7,7.1)	0.02
Meat						
No (*n =* 458)	—	—		—	—	
Yes (*n =* 282)	2.1	(−1.0, 5.2)	0.19	0.8	(−2.2, 3.7)	0.61
Egg						
No (*n =* 683)	—	—		—	—	
Yes (*n =* 57)	1.4	(−4.3, 7.0)	0.63	−0.1	(−5.5, 5.2)	0.96

1 Data shown are the coefficient B with 95% confidence interval (CI) and *P* value based on Tobit regression.

2 Adjusted for age (continuous) and sex.

3 Adjusted for age (continuous), sex, and elevated serum C-reactive protein and α_1_-acid glycoprotein, categorized as shown.

Interestingly, serum RBP was strongly positively associated with serum IGF-1 (Table 5). With <1.05 μmol/L as reference, serum RBP <0.70 and 0.70–1.05 μmol/L were associated with 14.3 (95% CI: 9.6, 19.1) and 6.1 (95% CI: 1.6, 10.6) μg/L lower serum IGF-1, respectively, after adjustment for age, sex, and inflammation. The findings did not change if the individual serum RBP was corrected for inflammation (rather than adjusting for inflammation in the Tobit regression analysis). Markers of iron, vitamin B12, and folate status were not associated with serum IGF-1, after adjustment for age, sex, and inflammation.

**TABLE 5:** Markers of micronutrient status as correlates of serum insulin-like growth factor-1 (μg/L) among 740 children aged 12–59 mo with stunting^1^.

	Age and sex adjusted^2^			Age, sex, and inflammation adjusted^3^			
	*B*	(95% CI)	*P*	*B*	(95% CI)	*P*	*P* for trend
Serum ferritin (μg/L)							0.84
<12 (*n =* 120)	−6.5	(−10.9, −2.1)	0.004	0.6	(−4.0, 5.2)	0.80	
12–24 (*n =* 143)	3.2	(−1.9, 8.2)	0.221	4.3	(−0.6, 9.2)	0.09	
>24 (*n =* 471)	—	—	—	—	—	—	
Serum soluble transferrin receptor (mg/L)							
<8.3 (*n =* 282)	—	—	—	—	—	—	—
>8.3 (*n =* 452)	−1.6	(−4.9, 1.6)	0.33	0.4	(−2.8, 3.5)	0.82	
Plasma cobalamin (pmol/L)							0.64
<148 (*n =* 25)	−1.2	(−9.8, 7.3)	0.78	−3.0	(−11.1, 5.2)	0.48	
149–222 (*n =* 144)	1.8	(−2.1, 5.7)	0.36	2.5	(−1.3, 6.2)	0.20	
>222 (*n =* 544)	—	—	—	—	—	—	
Plasma methylmalonic acid (μmol/L)							0.85
<0.45 (*n =* 482)	—	—	—	—	—	—	
0.45–0.75 (*n =* 129)	0.2	(−3.9, 4.2)	0.94	1.2	(−2.7, 5.1)	0.60	
>0.75 (*n =* 116)	−2.1	(−6.4, 2.1)	0.32	−0.9	(−4.9, 3.2)	0.68	
Plasma folate (nmol/L)							0.22
<20 (*n =* 60)	1.5	(−4.3, 7.2)	0.61	4.0	(−1.5, 9.4)	0.15	
20–30 (*n =* 203)	−1.4	(−5.0, 2.1)	0.43	0.6	(−2.8, 4.0)	0.74	
>30 (*n =* 423)	—	—	—	—	—	—	
Serum retinol-binding protein (μmol/L)							<0.001
<0.70 (*n =* 338)	−17.5	(−22.1, −12.8)	<0.001	−14.3	(−19.1, −9.6)	<0.001	
0.70–1.05 (*n =* 309)	−5.8	(−10.5, −1.1)	0.02	−6.1	(−10.6, −1.6)	0.009	
>1.05 (*n =* 87)	—	—	—	—	—	—	

1 Data shown are the coefficient B with 95% confidence interval (CI) and *P* value based on Tobit regression.

2 Adjusted for age (continuous) and sex.

3 Adjusted for age (continuous), sex, and elevated serum C-reactive protein and α_1_-acid glycoprotein, categorized as shown.

Sensitivity analysis excluding the 56 children with serum IGF-1 below the limit of detection gave similar estimates and findings.

## Discussion

As expected, serum IGF-1 was low among stunted children, and strongly associated with the severity of stunting. In addition, it showed a stronger association FFM than FM as in other studies [11,[25], [26], [27]]. Indeed, based on data from the MAGNUS trial, we previously reported [21] that unsupplemented stunted children accumulate FM at the expense of FFM, whereas supplementation increased serum IGF-1, linear catch-up growth, and accretion of FFM rather than FM [21]. This may reflect the important role of serum IGF-1 in regulating linear growth and body composition in young children [28]. Because height and body composition change slowly over time and are difficult to measure with precision, serum IGF-1 may have advantages as an outcome in studies assessing correlates of child growth.

### Infections and inflammation

The low serum IGF-1 was partly explained by elevated serum concentrations of the acute-phase reactants, which were seen in more than half of the children. A positive malaria test and reported fever, but not cough, were associated with low serum IGF-1, but these associations appeared entirely mediated by inflammation. In contrast, the strong association between current diarrhea and low serum IGF-1 was maintained after adjusting for inflammation. Our findings are in accordance with those from a longitudinal study among young children in Zimbabwe [29]. On the basis of path analysis, the study found that the associations of cough and fever with low serum IGF-1 were mediated by the acute-phase response. In contrast, the association between diarrhea and serum IGF-1 was not, but likely explained by gastrointestinal loss of nutrients of importance for serum IGF-1 [29]. A study from Malawi [30] found that children with malaria parasitemia or positive for *Campylobacter*, *Shigella,* and *Enterovirus*, but neither *Cryptosporidium* nor *Giardia*, had lower serum IGF-1. A path analysis suggested that apart from *Enterovirus*, these associations were mediated through systemic inflammation.

Thus, it is likely that intestinal infections with no systemic inflammation mainly reduce serum IGF-1 through reduced absorption and increased excretion of nutrients important to IGF-1 synthesis. As for infections accompanied by systemic inflammation, it is possible that there is a direct downregulation of serum IGF-1. Indeed, downregulation of serum IGF-1 may be a part of the acute-phase response, as the resulting transient cessation of growth may save energy and nutrients needed for defense against invading pathogens. However, in addition to possibly directly downregulating serum IGF-1, the acute-phase response also reduces intake and absorption, as well as increases utilization and loss of nutrients that may also be of importance to the synthesis of IGF-1. For example, it has been shown that considerable amounts of vitamin A bound to RBP are excreted in urine during infections, including acute diarrhea [31,32]. It may be speculated that IGF-1, which at 7649 Daltons is only about half the size of RBP, is also lost through urine during infections with systemic inflammation, and through the gastrointestinal tract in diarrhea.

### Foods and nutrients

The nutrients most likely to be essential to serum IGF-1 synthesis and growth are type II or growth nutrients, including amino acids, zinc, phosphorus, among others [8,15,18]. However, dietary intake of specific nutrients is difficult to assess, and bioavailability varies greatly, depending on a number of factors. Furthermore, growth nutrients are characterized by a lack of body stores and, hence, valid markers of status, so the roles of these nutrients in relation to serum IGF-1 and growth are difficult to study [18]. The effects of growth nutrients are best studied in nutrient intervention trials, whereas it is more feasible to study associations between types of foods (milk and other animal-source foods) and serum IGF-1, as well as markers of type 1 nutrient status and serum IGF-1, based on observational studies.

#### Milk

We found that intake of milk, but not meat or eggs, within the previous 24 h was associated with higher serum IGF-1. Given the considerable day-to-day variation in intake of animal-source foods, these findings should be taken with caution. Furthermore, we are unable to establish causality based on a cross-sectional study. Even if associations between intake of animal-source foods and serum IGF-1 are causal, the direction cannot be established, as it is also possible that illness resulting in low serum IGF-1 and poor growth could affect intake of animal-source foods. However, an effect of milk intake on serum IGF-1 would be in accordance with the conclusion of a meta-analysis of observational studies and randomized trials [33], as well as several later studies [27,34,35]. For example, a trial among children in Ghana found that skimmed milk (providing 8.8 g milk protein/d) given in a porridge increased serum IGF-1, but the same amount of protein with only half from milk and half from rice did not [27]. Despite the effects of skimmed milk on serum IGF-1 [27,[33], [34], [35]], neither milk protein nor whey permeate (containing lactose and milk minerals) had effects on linear growth or serum IGF-1 in the MAGNUS trial, from which the baseline data for this study were available [21]. The MAGNUS trial was a 2×2 factorial trial designed to assess which of the individual components of skimmed milk, were responsible for the effects seen. To keep protein and energy constant, milk protein (around 7 g/d) was compared with soy isolate protein, and whey permeate was compared with maltodextrin.

If indeed the association between milk intake and serum IGF-1 in our study reflects causality, a possible explanation for the lack of effect of milk protein on growth in the MAGNUS trial could be that the comparator for milk protein was soy isolate, a high-quality protein with no or minimal antinutrients [21]. Alternatively, it could be that the effect of milk is attributable to its zinc content, and that zinc was maintained in neither the milk protein nor the whey permeate. Whether zinc is maintained in the protein fraction depends on the pH during the process, and could be lacking from both milk components.

#### Micronutrients

We found that low vitamin A status was associated with lower serum IGF-1. Although vitamin A is not a growth nutrient like protein and zinc, it has been shown to be involved in GH gene expression [19] and regulation of nocturnal GH secretion [36]. Yet, a meta-analyses based on 19 data sets from intervention trials among children <5 y found no evidence of the effect of vitamin A on linear growth [37]. A later meta-analysis among children from 2 to 14 y, based on 16 data sets from 5 trials, found small, inconsistent effects of vitamin A on linear growth [38].

Even if vitamin A is essential to GH/IGF-1 synthesis, vitamin A supplementation trials may not reveal effects on linear growth unless baseline vitamin A status is low. Furthermore, if vitamin A status is very low, it is likely that the intake and bioavailability of zinc and other growth nutrients will be low as well. Hence, a vitamin A intervention may not increase serum IGF-1 and lead to a growth spurt if zinc or other growth nutrients are limiting. And vitamin A supplementation may upregulate GH/IGF-1, but lack of zinc and other growth nutrients may limit catch-up of linear growth.

Vitamin A is essential to epithelial integrity and various immune functions [39]. Hence, deficiency increases risk of infectious disease morbidity, such as diarrhea and respiratory tract infections [39]. Therefore, the effect of vitamin A supplementation on linear growth shown in a few trials [38] may be mediated through reduction in infectious diseases and inflammation rather than through synthesis of serum IGF-1 per se. Because our study was cross-sectional, inflammation is a potential confounder because it downregulates both serum RBP and serum IGF-1. Importantly, the association we found remained after adjustment for inflammation, as well as after correction for the inflammation of individual serum RBP measurements.

None of the markers of iron, cobalamin, or folate status were associated with serum IGF-1. Although iron deficiency is widespread and contributes to anemia, it contributes little to infectious disease mortality [40] and growth faltering [37]. Cobalamin and folate are essential for DNA synthesis, but there is hardly any data on their role in stunting. A cobalamin supplementation trial among young children in India found effects on linear growth in subgroups of children with stunting or wasting [41].

A strength of our study is the large sample size, which provides high precision around estimates and, consequently, high power to detect differences between groups. The high follow-up rate reduces the potential for selection bias. It is also a strength that we have data on markers of 4 different micronutrients, including both serum Fe and serum sTfR (iron), and plasma Cob and plasma MMA (B12), as well as data on body composition, based on a stunting-specific equation [23]. Among the limitations are the cross-sectional design, preventing us from making causal inferences, and the fact that we only have data on intake of animal-source foods based on 24-h recall and data on morbidity based on only maternal recall, and malaria based on an antigen-test only.

In conclusion, milk intake and vitamin A status were positively associated with serum IGF-1. In contrast to milk, vitamin A has not consistently been associated with growth. This requires further investigation.

## Authors contributions

The authors’ responsibilities were as follows – EMu, HF, BG: designed the research; EMa, NEN, JM, RM, EMu, HF, BG: conducted the research; EMa, NEN, HF, BG, CR: performed statistical analysis; EMa, NEM, HF, BG, EMu, SF, AB, CM, KFM: interpreted the data; HF: wrote the article; BG: primary responsibility for final content; and all authors: read and approved the final manuscript.

## Funding

This research was funded by Arla Food for Health, a public–private research partnership between the University of Copenhagen, Aarhus University and the dairy company, Arla. Additional funds were obtained from the Danish Dairy Research Foundation, Augustinus Fonden, Læge Sofus Carl Emil Friis og Hustru Olga Doris Friis’ Legat, and A. P. Møller Fonden til Lægevidenskabens Fremme. The funding sources were not involved in study design, data collection, analysis, interpretation, or decision to publish.

## Conflict of interest

The authors report no conflicts of interest.
